# Economic evaluation of chemoprevention of breast cancer with tamoxifen and raloxifene among high-risk women in Japan

**DOI:** 10.1038/sj.bjc.6604869

**Published:** 2009-01-13

**Authors:** M Kondo, S-L Hoshi, M Toi

**Affiliations:** 1 Department of Health Care Policy and Management, Graduate School of Comprehensive Human Sciences, University of Tsukuba, 1-1-1 Tennoudai, Tsukuba, Ibaraki 305-8577, Japan; 2 Clinical Research Division, Tokyo Metropolitan Cancer and Infectious Disease Centre, Komagome Hospital, 3-18-22 Honkomagome, Bunkyo-ku, Tokyo 113-8677, Japan; 3 Department of Surgery, Graduate School of Medicine, Kyoto University, 54 Kawaracho, Shogoin, Sakyo-ku, Kyoto 606-8507, Japan

**Keywords:** breast cancer, chemoprevention, cost-effectiveness, prophylaxis, raloxifene, tamoxifen

## Abstract

Raloxifene was approved for chemoprevention against breast cancer among high-risk women in addition to tamoxifen by the US Food and Drug Administration. This study aims to evaluate cost-effectiveness of these agents under Japan's health system. A cost-effectiveness analysis with Markov model consisting of eight health states such as healthy, invasive breast cancer, and endometrial cancer is carried out. The model incorporated the findings of National Surgical Adjuvant Breast and Bowel Project P-1 and P-2 trial, and key costs obtained from health insurance claim reviews. Favourable results, that is cost saving or cost-effective, are found by both tamoxifen and raloxifene for the introduction of chemoprevention among extremely high-risk women such as having a history of atypical hyperplasia, a history of lobular carcinoma *in situ* or a 5-year predicted breast cancer risk of ⩾5.01% starting at younger age, whereas unfavourable results, that is ‘cost more and gain less’ or cost-ineffective, are found for women with a 5-year predicted breast cancer risk of ⩽5.00%. Therapeutic policy switch from tamoxifen to raloxifene among postmenopausal women are implied cost-effective. Findings suggest that introduction of chemoprevention targeting extremely high-risk women in Japan can be justifiable as an efficient use of finite health-care resources, possibly contributing to cost containment.

Several clinical trials have demonstrated the effectiveness of prophylactic administration of selective oestrogen receptor modulators (SERMs) such as tamoxifen (Fisher *et al*, 2005; Cuzick *et al*, 2007; Powles *et al*, 2007; Veronesi *et al*, 2007b) and raloxifene (Cauley *et al*, 2001; Martino *et al*, 2004; Vogel *et al*, 2006) in reducing incidence of breast cancer among women at high risk of developing the disease. Tamoxifen was approved for prophylaxis by the US Food and Drug Administration in 1998, and raloxifene was also approved for postmenopausal women in 2007.

Tamoxifen reduces the risk of breast cancer whereas increasing the risk of adverse events such as endometrial cancer and pulmonary embolism. Raloxifene is a second-generation SERM usually used for osteoporosis treatment, and it reduces the risk of invasive breast cancer with a lower risk of known adverse events associated with SERMs, compared to tamoxifen. This is because raloxifene does not induce the unwanted stimulation of endometrium (Delmas *et al*, 1997). Therefore, raloxifene is considered to have a better clinical property as prophylactic agent, although it is inferior to tamoxifen in preventing noninvasive breast cancer. More women at high risk of developing breast cancer are expected to take raloxifene as their breast cancer prevention drug in the United States (Bevers, 2007).

However, both of these agents have been neither approved nor made available for its use as breast cancer prevention in Japan, although experts have shown their expectations (Iwata and Saeki, 2006). It is said that there are five hurdles to overcome in addressing intervention in the diffusion process of new drug: quality, safety, efficacy, cost-effectiveness, and affordability (Trueman *et al*, 2001). This paper aims to present evidence to the fourth hurdle, cost-effectiveness of both agents, under Japan's health system. Although cost-effectiveness of prophylactic use of tamoxifen has been reported from the USA (Noe *et al*, 1999; Grann *et al*, 2000; Smith and Hillner, 2000; Hershman *et al*, 2002; Melnikow *et al*, 2006) and Australia (Eckermann *et al*, 2003), that of raloxifene has not been published to date except as a part of economic evaluation of osteoporosis management (Armstrong *et al*, 2001; Kanis *et al*, 2005). This paper also simulates a therapeutic policy switch from tamoxifen to raloxifene among postmenopausal women to illustrate the relative value of raloxifene. Consequently, it should have implications to the developed countries where chemoprevention with tamoxifen is already in practise.

## Methods

We conduct a cost-effectiveness analysis with Markov modelling based on the findings of the National Surgical Adjuvant Breast and Bowel Project (NSABP) P-1 trial (Fisher *et al*, 2005), the NSABP P-2 trial (Vogel *et al*, 2006), and the literature on costing under Japan's health system including sensitivity analyses from societal perspective. Although longer follow-up results for tamoxifen are reported from the first International Breast Cancer Intervention Study (IBIS-I; Cuzick *et al*, 2007) and the Royal Marsden trial (Powles *et al*, 2007), NSABP P-1 trial with a shorter follow-up period is chosen as clinical evidence for our modelling to make clear comparisons with NSABP P-2 trial of raloxifene. The long-term outcomes for tamoxifen (Veronesi *et al*, 2007a) are considered in our sensitivity analyses. We use TreeAge Pro 2008 (TreeAge Software Inc.) for our economic modelling.

### High-risk women

We model high-risk women according to the risk classifications featured in the report of clinical trials: three levels (⩾1.66, 3.01–5.00%, ⩾5.01%) of a 5-year predicted breast cancer risk, with a history of lobular carcinoma *in situ* (LCIS), and with a history of atypical hyperplasia (AH). A 5-year predicted breast cancer risk of an individual woman used in the trials is based on Gail *et al* model 2 (Gail and Costantino, 2001), which is validated for white women (Rockhill *et al*, 2001) and African American women (Gail *et al*, 2007), to date. We assume the same model is good for Japanese women.

We also model the ages of starting prophylaxis: 35, 50, 60 years old for tamoxifen, and 50, 60 years old for raloxifene taking the menopause into account.

### Markov model

**Figure 1:**
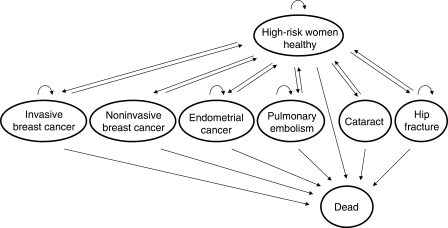
Markov model.

We construct a Markov model of courses followed by high-risk women, which is shown in Figure 1. Eight health states are modelled according to clinical events monitored and found significant in P-1 trial and P-2 trial: (1) healthy; (2) invasive breast cancer; (3) noninvasive breast cancer, (4) endometrial cancer; (5) pulmonary embolism; (6) cataract; (7) hip fracture; and (8) dead. Healthy women at high risk of the disease, women with invasive and noninvasive breast cancer are the target health states for chemoprevention. An increase in risk of endometrial cancer, pulmonary embolism, and cataract are known as adverse effects of SERMs, whereas a decrease in risk of hip fracture is known as a beneficial effect. Transitions between health states are indicated with arrows.

The time span of each stage is set at 1 year, since trials report annual incidence rates. Markov process is repeated until death or age 100, whichever comes first, since all events are expected to occur within this time horizon. Women who survive after the age of 100 years are assumed to die regardless of breast cancer development.

### Chemoprevention

Prophylaxis with SERMs is continued for 5 years, or discontinued in case of adverse events, which is similar to the regimen employed in clinical trials.

### Comparisons

We compare outcomes and costs in terms of incremental cost-effectiveness ratios (ICERs) between *status quo* in Japan, without prophylaxis, and hypothetical practise, with prophylaxis, by the agent (tamoxifen and raloxifene), the risk classification, and the age of starting prophylaxis. 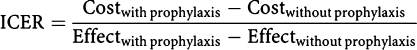 We also compare prophylaxis with tamoxifen and prophylaxis with raloxifene to estimate the relative value of raloxifene to tamoxifen, although this does not depict any marginal change in Japan.

### Outcome estimation

Outcomes in terms of life years gained (LYGs) and quality adjusted life years (QALYs) are estimated by assigning transitional probabilities and utility weights to Markov model from the literature.

Transitional probabilities from healthy state to disease states in Markov model are shown in Table 1 according to the findings from the clinical trials. Risk reduction effect of SERMs is assumed to continue during the 5-year course of prophylaxis.

Table 2 summarises other assumptions such as transitional probabilities from disease states to dead state and utility weights used in Markov model. The share of clinical stages of invasive breast cancer at diagnosis are adopted from a nationwide survey on breast cancer screening (Japan Cancer Society, 2007), of which prognosis is calculated from corresponding follow-up cases at Tokyo Metropolitan Cancer and Infectious Disease Centre Komagome Hospital. The prognosis of endometrial cancer is also adopted from a nationwide cancer registry (Japanese Society of Obstetrics and Gynecology, 2000). The prognosis of pulmonary embolism and hip fracture are taken from Sakuma *et al* (2004); Kitamura *et al* (1998), respectively. Japanese female population mortality rates from Vital Statistics (Ministry of Health, Labour and Welfare, 2005a) are applied for other transitions to dead state.

It is more preferable to adopt utility weights from a consistent study that assesses our six disease states in Japan, but there is no Japanese utility weight in the literature to date, which may be applied to any health states in our model. To illustrate the typical patient states, we adopt the weights assessed in developed countries considering them as the best available knowledge, and choosing them under the consensus of staff doctors at Tokyo Metropolitan Cancer and Infectious Disease Centre Komagome Hospital (de Koning *et al*, 1991; Hillner *et al*, 1993; Smith and Hillner, 1993; Grann *et al*, 1998; Earle *et al*, 2000; Armstrong *et al*, 2001; Chau *et al*, 2003; Cykert *et al*, 2004; Naeim and Keeler, 2005; Ruof *et al*, 2005).

Outcome is discounted at a rate of 3%.

### Costing

From societal perspective, costing should cover the opportunity cost borne by various economic entities in the society. In the context of this study, costs borne by women or third party payers including the government and social insurers are considered, although there is no particular assumption about who bears the cost of chemoprevention. According to the national medical care fee schedule, the amount of direct payments to health-care providers is estimated as cost, whereas costs to sectors other than health and productivity losses are left uncounted.

Health states are identified as cost items in Markov model. Table 3 summarises the cost of each health states. Being in healthy state, women with chemoprevention take 20 mg per day, ¥82.6 (£0.41; £1=¥200), of tamoxifen, or 60 mg per day, ¥148.5 (£0.74), of raloxifene, prescribed regularly for 5 years, and annual mammography checkup. Women without chemoprevention also undergo annual mammography checkup. Although the state is labelled as ‘healthy’, it includes all other diseases that are not modelled in Markov model. Annual treatment costs by the age stratum are approximated by annual health-care expenditure per woman adopted from National Health-Care Expenditure (Ministry of Health, Labour and Welfare, 2005b). As it is well known that the cost of health care in the last year of life tends to be large, these are shown separately after an adjustment based on Fukawa (1998).

Table 3 also summarises the treatment cost of invasive breast cancer by the age stratum. In the case of cancer care, the cost in the first year after diagnosis tends to be large as well as in the last year of life, so here again, the costs are shown separately. These figures are obtained from insurance claim reviews at Tokyo Metropolitan Cancer and Infectious Disease Centre Komagome Hospital. As to the cost of the first year, recent breast cancer cases of stage I and stage II that have undergone initial treatment with a follow-up of 1 year are retrospectively selected so that each age strata has 40 cases. As to the yearly cost of the second year and thereafter, 40 cases for each age strata are randomly selected from follow-up cases initially diagnosed as stage I and stage II. As to the cost of the last year of life, recent 80 fatal cases are retrospectively selected, as the number of these is relatively limited. Insurance claims of these total of 400 cases for 1 year are reviewed to calculate average annual costs by the age strata. Then an adjustment is made to include the cost of prescription to be filled at external pharmacies, such as in the case of adjuvant hormonal therapy, which is based on the consensus among staff doctors.

Costs of disease states are summarised in Table 3 as well. Treatment costs of noninvasive breast cancer, endometrial cancer, cataract, and hip fractures are adopted from a background study for the development of Japanese prospective payment system to health-care providers, diagnosis procedure combination (Matsuda and Ishikawa, 2003), whereas treatment cost of pulmonary embolism is adopted from Fuji *et al* (2005).

Costs are also discounted at a rate of 3%.

### Sensitivity analyses

To deal with the uncertainty of probabilities, utility weights, and costs used in our economic model, one-way sensitivity analyses are performed. Transitional probabilities from healthy state to disease states shown in Table 1 are varied in 1.5 times of 95% confidence intervals (CI) reported from the clinical trials. 95% CI is often used for similar exercises of sensitivity analyses, but we set wider range for the applicability of the clinical trial data to Japanese women. The other probabilities shown in Table 2 are changed by ±50%. Utility weights are changed by ±20%, and we think this could cover the difference between the utility weights of Japanese women and those of the other developed nations. Costs shown in Table 3 are changed by ±50%. Discount rate is also changed from 0 to 6%.

Acknowledging the long-term outcomes for tamoxifen in the IBIS-I trial (Cuzick *et al*, 2007) and the Royal Marsden trial (Powles *et al*, 2007), risk reduction effect of tamoxifen is prolonged from 5 to 10 and 15 years without any risk increase of adverse events after the completion of prophylaxis.

## Results

### Outcomes

Table 4 shows the results of cost-effectiveness analysis comparing prophylaxis with no prophylaxis.

In the comparison between prophylaxis with tamoxifen *vs* no prophylaxis, most outcomes in terms of LYGs are increased by chemoprevention except for women with a 5-year predicted breast cancer risk of ⩾1.66% starting at age 50, and women with a 5-year predicted breast cancer risk of 3.01–5.00% starting at age 50 and 60. Outcomes in terms of QALYs are also increased except for women with a 5-year predicted breast cancer risk of ⩾1.66% starting at age 50 and 60, women with a 5-year predicted breast cancer risk of 3.01–5.00%, and women with a history of LCIS starting at age 60. The largest outcome gain in terms of QALYs, 0.105, is estimated among women with a history of AH starting at age 35.

Between prophylaxis with raloxifene *vs* no prophylaxis, all outcomes in terms of LYGs are increased by chemoprevention. Outcomes in terms of QALYs are increased except for women with a 5-year predicted breast cancer risk of ⩾1.66%, and women with a 5-year predicted breast cancer risk of 3.01–5.00%. The largest outcome gain in terms of QALYs, 0.058, is estimated among women with a history of AH starting at age 50.

Table 5 shows the results of cost-effectiveness analysis of therapeutic policy switch from tamoxifen to raloxifene.

Raloxifene is consistently superior to tamoxifen across presented risk classifications and starting ages of prophylaxis.

### Costs

In the comparison between prophylaxis with tamoxifen *vs* no prophylaxis (Table 4), cost savings are estimated in higher risk classifications, among women with a history of LCIS or AH, starting at younger age. The largest saving, ¥367 901 (£1840), is estimated among women with a history of AH starting at age 35.

Between prophylaxis with raloxifene *vs* no prophylaxis, prophylaxes are found more costly. A cost saving of ¥10 387 (£52) is estimated among women with a history of AH starting at age 50.

When considering the therapeutic policy switch (Table 5), the use of raloxifene is consistently more costly than tamoxifen, as anticipated by the difference in price of agents.

### Cost-effectiveness

There is a suggested criterion for cost-effectiveness in Japan (Ohkusa, 2003) to be ¥6000 000 (£30 000) for one QALY gain, and both Tables 4 and 5 report judgements with this criterion.

In the comparison between prophylaxis with tamoxifen *vs* no prophylaxis, favourable results, that is ‘cost less and gain more’ or cost-effective, are obtained in higher risk classifications starting at younger age. Those are: women with a history of AH regardless of starting age, women with a history of LCIS starting at age 35 and 50, and women with a 5-year predicted breast cancer risk of ⩾5.01% starting at age 35 and 50.

Similar results are found between prophylaxis with raloxifene *vs* no prophylaxis. Favourable results are: women with a history of AH regardless of starting age, women with a history of LCIS starting at age 50, and women with a 5-year predicted breast cancer risk of ⩾5.01% starting at age 50.

As shown in Table 5, ICERs for the therapeutic policy switch of prophylactic agent from tamoxifen to raloxifene varies from ¥1839 670 per QALY (£9198 per QALY) to ¥6771 100 per QALY (£33 856 per QALY). The larger ICER is yet still close to the suggested criterion of ¥6000 000 per QALY (£30 000 per QALY).

### Stability of cost-effectiveness

One-way sensitivity analyses produce similar results across the agents, the risk classifications and the ages of starting prophylaxis. Therefore, we draw a cost-effectiveness plane to show the comparison between prophylaxis with raloxifene *vs* no prophylaxis among three risk classifications as an example: women with a 5-year predicted breast cancer risk of ⩾5.01%, women with a history of LCIS, and women with a history of AH.

**Figure 2:**
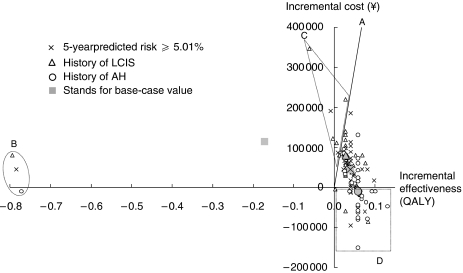
Illustration of key results of sensitivity analyses: prophylaxis with raloxifene *vs* no prophylaxis starting at age 50.

Figure 2 plots three base-case values and 306 results (102 changes of variables × three different risk classifications). Line OA indicates the threshold of favourable ICER compared to the suggested criterion of ¥6000 000 (£30 000) for one QALY gain. Most results are plotted close to base-case value, which suggest the stability of our model. Results for women with a history of AH remain constantly favourable being cost saving or cost-effective by the change of variables except for one plot shown as in area B. However, several results for women with a 5-year predicted breast cancer risk of ⩾5.01% and for women with a history of LCIS cross the threshold line, the vertical axis or the horizontal axis from the base-case values. Three plots in area B and seven plots in area C indicate that results turn unfavourably, that is cost-ineffective or ‘gain less’, whereas plots in area D show that results become cost saving.

Our model is most sensitive to the utility weight for healthy state under chemoprevention, of which plots are drawn in area B. Its change to 0.79 turns incremental effectiveness into negative. Critical values to change the judgement are 0.98, which makes the ICERs of women with a 5-year predicted breast cancer risk of ⩾5.01% and woman with a history of LCIS cost-ineffective, and the value of 0.96 makes women with a history of AH ‘gain less’. The model is also sensitive to the discount rate, of which plot is drawn in area C. Its raise of 5.9 and 4.3% makes the ICERs of women with a 5-year predicted breast cancer risk of ⩾5.01% and women with a history of LCIS cost-ineffective, respectively. The cost of chemoprevention is also influential to the results, of which results are shown in areas C and D. A price increase of more than 30% for raloxifene makes the ICER of women with a history of LCIS cost-ineffective, whereas a price decrease of more than 16 or 29% make the results for women with a 5-year predicted breast cancer risk of ⩾5.01% and women with a history of LCIS cost saving, respectively. Changes of the probabilities of transition to invasive breast cancer, endometrial cancer, and hip fracture are also plotted in areas C and D. Raising the probability of invasive breast cancer beyond 0.00710 and 0.00683 makes the ICERs of women with a 5-year predicted breast cancer risk of ⩾5.01% and women with a history of LCIS cost-ineffective, whereas lowering to less than 0.00456 or 0.00436 make the results for women with a 5-year predicted breast cancer risk of ⩾5.01% and women a history of LCIS cost saving, respectively. Raising the probability of endometrial cancer beyond 0.00369 and 0.00271 makes the ICERs of women with a 5-year predicted breast cancer risk of ⩾5.01% and women with a history of LCIS cost-ineffective, respectively. Raising probability of hip fracture beyond 0.00098 makes the results for women with a history of LCIS cost saving. The other plots in area C reflect a raise of utility weight for invasive breast cancer after the second year.

Prolonging risk reduction effect of tamoxifen from 5 to 10 and 15 years without any risk increase of adverse events after the completion of prophylaxis brings more favourable results. For example, the effect of 10 years results in ‘cost less and gain more’ for every risk classification starting at age 35, whereas the effect of 15 years makes no change in the results of ‘cost more and gain less’ among women with a 5-year predicted breast cancer risk of ⩾1.66% starting at age 50 and 60.

## Discussion

We conduct a cost-effectiveness analysis of SERMs as prophylactic agents against breast cancer among high-risk women by making comparisons between *status quo* in Japan, without prophylaxis, and hypothetical practise, with prophylaxis, by the agent (tamoxifen and raloxifene), the risk classification, and the age of starting prophylaxis.

We find that prophylaxis with tamoxifen results in ‘cost less and gain more’ among extremely high-risk women such as those with a 5-year predicted breast cancer risk of ⩾5.01%, those with a history of LCIS, and those with a history of AH starting at age 35 and 50. Prophylaxis with raloxifene is also found ‘cost less and gain more’ for women with a history of AH starting at age 50. The younger the age of starting prophylaxis, the more the cost saving and outcome gain. We also find that prophylaxis with tamoxifen for women with a history of AH starting at age 60 results in favourable ICER compared to the suggested criterion of ¥6000 000 (£30 000) for one QALY gain. Prophylaxis with raloxifene is also found cost-effective for women with a 5-year predicted breast cancer risk of ⩾5.01% starting at age 50, those with a history of LCIS starting at age 50 and those with a history of AH starting at age 60. The younger the age of starting prophylaxis, the more favourable the ICER. Within the same risk classification and starting age, raloxifene tends to gain more and cost more compared to tamoxifen. On the contrary, we also find that prophylaxes with tamoxifen or raloxifene for women with a 5-year predicted breast cancer risk of ⩽5.00% tend to result in ‘cost more and gain less’.

These findings are similar to the previous economic evaluations of chemoprevention of breast cancer with tamoxifen including analyses of risk level differences such as Noe *et al* (1999); Grann *et al* (2000); Hershman *et al* (2002); Melnikow *et al* (2006), although these studies are carried out under the US health system.

Our findings suggest that introduction of chemoprevention with SERMs targeting extremely high-risk women in Japan can be justifiable as an efficient use of finite health-care resources, possibly contributing to cost containment. The cost saving results suggest chemoprevention not only cost-effective but also affordable. Taking the superiority of raloxifene in outcome gain and the difference in indication into account, it is recommendable to administer tamoxifen for premenopausal women and raloxifene for postmenopausal women.

Our economic model is found sensitive to the utility weight for healthy state under chemoprevention, the discount rate and the cost of chemoprevention, in addition to the probabilities of transition to invasive breast cancer, endometrial cancer, or hip fracture. This is anticipated because these variables are supposed to influence the cost-effectiveness of preventive services. We think that our economic model succeeds in explaining the context under consideration.

We also analysed the cost-effectiveness of therapeutic policy switch of agent, tamoxifen to raloxifene among postmenopausal women, although this does not depict any marginal change in Japan. All simulated ICERs by risk classifications starting at age 50 and 60 fall in a favourable level. Due caution is needed in transferring these findings from our Japanese model to other health system (Drummond and Pang, 2001), but it implies that the administration of raloxifene instead of tamoxifen for postmenopausal high-risk women could be economically acceptable in developed countries where chemoprevention with tamoxifen is already in practise.

There are a couple of points to consider when interpreting our results. Our model depends on clinical evidence established in the United States by P-1 and P-2 trial. Composition of ethnicity and life styles of participating women are different from those of Japanese women. This also relates to another point, that is the validity of the 5-year risk prediction model defining high-risk women. As already mentioned in Methods section, it is based on Gail *et al* model 2 (Gail and Costantino, 2001), which has been validated for white women (Rockhill *et al*, 2001) and African American women (Gail *et al*, 2007) only. Our approach is acceptable as to these points, as the results of P-1 and P-2 trial are the best available evidence to date for the objectives of this study, and similar risk factors to Gail *et al* model 2 are identified in a model of individualised probability of developing breast cancer for Japanese women (Ueda *et al*, 2003), and the function of ethnic difference in developing breast cancer is reported as small (Chen *et al*, 2004). Our model also depends on utility weights reported from Western countries, as none of those from Japan are available. However, our findings of consistent outcomes in terms of LYGs offer reasonable conclusions.

In summary, this study suggests that chemoprevention of breast cancer with SERMs targeting high-risk women such as a 5-year predicted breast cancer risk of ⩾5.01%, women with a history of LCIS, and women with a history of AH, clears the hurdles of introducing new intervention by means of cost-effectiveness and affordability, with best available evidence. Although further studies and policy formulations are necessary about breast cancer chemoprevention in Japan, this study also implies that the administration of raloxifene instead of tamoxifen may be cost-effective under the context of developed countries where chemoprevention with tamoxifen has already been adopted.

## Figures and Tables

**Table 1:** Transitional probabilities from healthy state to disease states in Markov model

	**Placebo**		**Tamoxifen**			**Raloxifene**		
	**Base-case value**	**Source**	**Base-case value**	**Range tested in sensitivity analysis^a^**	**Source**	**Base-case value**	**Range tested in sensitivity analysis^a^**	**Source**
*Invasive breast cancer*								
Five-year predicted breast cancer risk ⩾1.66%								
Age of starting prophylaxis								
35	0.00632	Fisher *et al* (2005)	0.00404	0.00235–0.00641	Fisher *et al* (2005)			
50	0.00587	Fisher *et al* (2005)	0.00333	0.00168–0.00573	Fisher *et al* (2005)	0.00310	0.00184–0.00490	Fisher *et al* (2005), Vogel *et al* (2006)
60	0.00668	Fisher *et al* (2005)	0.00330	0.00165–0.00567	Fisher *et al* (2005)	0.00366	0.00213–0.00585	Fisher *et al* (2005), Vogel *et al* (2006)
Five-year predicted breast cancer risk 3.01–5.00%	0.00451	Fisher *et al* (2005)	0.00270	0.00108–0.00534	Fisher *et al* (2005)	0.00203	0.00101–0.00349	Fisher *et al* (2005), Vogel *et al* (2006)
Five-year predicted breast cancer risk ⩾5.01%	0.01198	Fisher *et al* (2005)	0.00515	0.00245–0.00893	Fisher *et al* (2005)	0.00561	0.00323–0.00894	Fisher *et al* (2005), Vogel *et al* (2006)
History of lobular carcinoma *in situ*	0.01170	Fisher *et al* (2005)	0.00627	0.00161–0.01476	Fisher *et al* (2005)	0.00614	0.00239–0.01226	Fisher *et al* (2005), Vogel *et al* (2006)
History of atypical hyperplasia	0.01042	Fisher *et al* (2005)	0.00255	0.00029–0.00686	Fisher *et al* (2005)	0.00286	0.00133–0.00523	Fisher *et al* (2005), Vogel *et al* (2006)
Noninvasive breast cancer	0.00012	Fisher *et al* (2005)	0.00004	0.00000–0.00652	Fisher *et al* (2005)	0.00006	0.00003–0.00009	Fisher *et al* (2005, Vogel *et al* (2006)
								
*Endometrial cancer*								
Age of starting prophylaxis								
35	0.00082	Fisher *et al* (2005)	0.00116	0.00010–0.00410	Fisher *et al* (2005)			
50 and 60	0.00058	Fisher *et al* (2005)	0.00308	0.00061–0.00992	Fisher *et al* (2005)	0.00194	0.00065–0.00403	Fisher *et al* (2005), Vogel *et al* (2006)
								
*Pulmonary embolism*								
Age of starting prophylaxis								
35	0.00013	Fisher *et al* (2005)	0.00025	0.00000–0.00420	Fisher *et al* (2005)			
50 and 60	0.00044	Fisher *et al* (2005)	0.00096	0.00020–0.00275	Fisher *et al* (2005)	0.00061	0.00028–0.00114	Fisher *et al* (2005), Vogel *et al* (2006)
Cataract	0.02285	Fisher *et al* (2005)	0.02775	0.02384–0.03206	Fisher *et al* (2005)	0.02192	0.01735–0.02734	Fisher *et al* (2005), Vogel *et al* (2006)
Hip fracture	0.00086	Fisher *et al* (2005)	0.00059	0.00022–0.00122	Fisher *et al* (2005)	0.00052	0.00016–0.00115	Fisher *et al* (2005), Vogel *et al* (2006)

a 1.5 times of 95% confidence interval.

**Table 2:** Assumptions used in Markov model

	**Assumption**	**Range tested in sensitivity analysis**	**Source**
*Transitional probabilities from disease states to dead state*			
Invasive breast cancer	0–9 years after diagnosis: prognosis of Japanese breast cancer patients by the stage	Change by±50%	Calculated from follow-up patients at Komagome Hospital
	Stage I: 0.0074, 0.0155, 0.0113, 0.0218, 0.0254, 0.0248, 0.0289, 0.0165, 0.01632		
	Stage II: 0.0054, 0.0474, 0.0570, 0.0334, 0.0398, 0.0321, 0.0275, 0.0295, 0.04672		
	(Proportions of stage at diagnosis are assumed stage I as 72% and stage II as 28%)	Change by±50%	Japan Cancer Society (2007)
	Thereafter: Japanese female population mortality rates	Change by±50%	Ministry of Health, Labour and Welfare (2005a)
Noninvasive breast cancer	Japanese female population mortality rates	Change by±50%	Ministry of Health, Labour and Welfare (2005a)
Endometrial cancer	0–4 years after diagnosis: prognosis of Japanese endometrial cancer patients 0.0660, 0.0546, 0.0328, 0.02813	Change by±50%	Japanese Society of Obstetrics and Gynecology (2000)
	Thereafter: Japanese female population mortality rates	Change by±50%	Ministry of Health, Labour and Welfare (2005a)
Pulmonary embolism	0 year after diagnosis: 0.08	Change by±50%	Sakuma *et al* (2004)
	Thereafter: Japanese female population mortality rates	Change by±50%	Ministry of Health, Labour and Welfare (2005a)
Cataracts	Japanese female population mortality rates	Change by±50%	Ministry of Health, Labour and Welfare (2005a)
Hip fracture	0–1 years after diagnosis: 0.11 and 0.19, respectively	Change by±50%	Kitamura *et al* (1998)
	Thereafter: Japanese female population mortality rates	Change by±50%	Ministry of Health, Labour and Welfare (2005a)
			
*Utility weights*			
Healthy	1.00	Change by±20%	
Healthy under chemoprevention for 5 years	0.99	Change by±20%	Smith and Hillner (1993), Hillner *et al* (1993), Naeim and Keeler (2005)
Invasive breast caner	0 year after diagnosis: 0.87, thereafter: 0.89	Change by±20%	de Koning *et al* (1991), Grann *et al* (1998)
Noninvasive breast cancer	0.98	Change by±20%	Earle *et al* (2000)
Endometrial cancer	0 year after diagnosis: 0.83, thereafter: 0.88	Change by±20%	Armstrong *et al* (2001), Cykert *et al* (2004)
Pulmonary embolism	0.70	Change by±20%	Chau *et al* (2003)
Cataract surgery	0.96	Change by±20%	Ruof *et al* (2005)
Hip fracture	0–1 years after diagnosis: 0.61 and 0.92, respectively	Change by±20%	Armstrong *et al* (2001)

**Table 3:** Costs (¥)

	**Healthy**			**Breast cancer**		
	**Base-case value**	**Range tested in sensitivity analysis**	**Source**	**Base-case value**	**Range tested in sensitivity analysis**	**Source**
*Chemoprevention*						
Tamoxifen	30 149	Change by±50%	Drug price list, etc			
Raloxifene	54 203	Change by±50%				
Prescription+annual mammography	44 980	Change by±50%				
Annual mammography	15 520	Change by±50%				
						
*Ages 35–49*						
First year after diagnosis				1978 064	Change by±50%	
Yearly cost				383 743	Change by±50%	
Ages 35–39	81 937	Change by±50%				
Ages 40–44	94 529	Change by±50%	Ministry of Health, Labour and Welfare (2005b), Fukawa (1998)			Insurance claim review
Ages 45–49	110 604	Change by±50%				
Terminal care cost, last year of life				5495 224	Change by±50%	
Ages 35–39	352 331	Change by±50%				
Ages 40–44	406 474	Change by±50%				
Ages 45–49	475 599	Change by±50%			Change by±50%	
						
*Ages 50–64*						
First year after diagnosis				2211 083	Change by±50%	
Yearly cost				542 857	Change by±50%	
Ages 50–54	151 625	Change by±50%	Ministry of Health, Labour and Welfare (2005b), Fukawa (1998)			Insurance claim review
Ages 55–59	195 085	Change by±50%				
Ages 60–64	258 723	Change by±50%				
Terminal care cost, last year of life				4106 271	Change by±50%	
Ages 50–54	651 986	Change by±50%				
Ages 55–59	838 866	Change by±50%				
Ages 60–64	1112 510	Change by±50%				
						
*Ages 65–79*						
First year after diagnosis				1530 259	Change by±50%	
Yearly cost				441 458	Change by±50%	
Ages 65–69	324 347	Change by±50%				
Ages 70–74	460 617	Change by±50%	Ministry of Health, Labour and Welfare (2005b), Fukawa (1998)			Insurance claim review
Ages 75–79	549 284	Change by±50%				
Terminal care cost, last year of life				3252 302	Change by±50%	
Ages 65–69	1394 690	Change by±50%				
Ages 70–74	1980 653	Change by±50%				
Ages 75–79	2361 923	Change by±50%				
						
*Ages 80+*						
First year after diagnosis			Ministry of Health, Labour and Welfare (2005b), Fukawa (1998)	961 181	Change by±50%	Insurance claim review
Yearly cost				185 151	Change by±50%	
Ages 80–84	576 290	Change by±50%				
Ages 85–89	647 941	Change by±50%				
Ages 90–94	557 429	Change by±50%				
Ages 95–100	465 059	Change by±50%				
Terminal care cost, last year of life				427 042	Change by±50%	
Ages 80–84	2478 049	Change by±50%				
Ages 85–89	2786 147	Change by±50%				
Ages 90–94	2396 943	Change by±50%				
Ages 95–100	1999 754	Change by±50%				
	**Diseases**					
	**Base-case value**		**Range tested in sensitivity analysis**		**Source**	
*Noninvasive breast cancer surgery, etc (DPC0900103x020xxx+ reimbursements by FFS)*	847 928		Change by±50%		Matsuda and Ishikawa (2003)	
						
*Endometrial cancer*						
Total hysterectomy, etc (DPC 1200203x01x0xx+ reimbursements by FFS)	1183 839		Change by±50%		Matsuda and Ishikawa (2003)	
*Pulmonary embolism*						
Total	469 890					
(Diagnosis)	(52 350)		Change by±50%		Fuji *et al* (2005)	
(Treatment)	(417 540)					
						
*Cataract*						
Surgery, etc (DPC 0201103x01x 000+reimbursements by FFS)	309 120		Change by±50%		Matsuda and Ishikawa (2003)	
*Hip fracture*						
Surgery, etc (DPC 1608003x02xx0x+ reimbursements by FFS)	1553 195		Change by±50%		Matsuda and Ishikawa (2003)	

DPC: diagnosis procedure combination; FFS: fee for service.

**Table 4:** Results of cost-effectiveness analysis (1)

	**CoCost (¥)**			**Effectiveness (LYGs)**			**Effectiveness (QALYs)**			**Incremental cost- effectiveness ratio**	
**No prophylaxis *vs* prophylaxis with tamoxifen**	**No prophylaxis**	**Tamoxifen**	**Incremental**	**No prophylaxis**	**Tamoxifen**	**Incremental**	**No prophylaxis**	**Tamoxifen**	**Incremental**	**(¥/LYG)**	**(¥/QALY)**
*Five-year predicted breast cancer risk ⩾1.66%*											
Starting at age 35	13 958 679	13 983 626	24 947	25.916	25.953	0.037	25.757	25.759	0.002	678 210	14 247 447
Starting at age 50	17 630 814	17 751 353	120 538	22.168	22.167	−0.001	22.040	22.000	−0.040	Cost more, gain less	Cost more, gain less
Starting at age 60	20 160 906	20 324 294	163 388	18.806	18.807	0.001	18.688	18.654	−0.034	120 849 008	Cost more, gain less
											
*Five-year predicted breast cancer risk 3.01–5.00%*											
Starting at age 35	13 627 472	13 685 368	57 896	26.005	26.035	0.030	25.879	25.872	−0.007	1 946 092	Cost more, gain less
Starting at age 50	17 579 407	17 732 900	153 493	22.195	22.185	−0.010	22.088	22.037	−0.051	Cost more, gain less	Cost more, gain less
Starting at age 60	20 251 937	20 444 141	192 203	18.808	18.797	−0.011	18.718	18.666	−0.052	Cost more, gain less	Cost more, gain less
											
*Five-year predicted breast cancer risk ⩾5.01%*											
Starting at age 35	14 956 349	14 667 969	−288 380	25.651	25.755	0.105	25.396	25.480	0.084	Cost less, gain more	Cost less, gain more
Starting at age 50	17 867 146	17 800 766	−66 379	22.049	22.096	0.047	21.832	21.854	0.022	Cost less, gain more	Cost less, gain more
Starting at age 60	19 958 433	20 058 020	99 857	18.797	18.825	0.028	18.614	18.618	0.004	3548 049	26 648 821
											
*History of lobular carcinoma* *in situ*											
Starting at age 35	14 908 314	14 717 649	−190 665	25.663	25.747	0.083	25.414	25.472	0.058	Cost less, gain more	Cost less, gain more
Starting at age 50	17 856 158	17 850 722	−5 386	22.054	22.085	0.031	21.841	21.843	0.002	Cost less, gain more	Cost less, gain more
Starting at age 60	19 968 466	20 093 211	124 745	18.798	18.815	0.017	18.618	18.606	−0.011	7282 700	Cost more, gain less
											
*History of atypical hyperplasia*											
Starting at age 35	14 687 003	14 319 102	−367 901	25.722	25.844	0.122	25.493	25.598	0.105	Cost less, gain more	Cost less, gain more
Starting at age 50	17 806 095	17 692 020	−114 075	22.079	22.139	0.060	21.884	21.922	0.038	Cost less, gain more	Cost less, gain more
Starting at age 60	20 015 243	20 096 731	81 488	18.800	18.837	0.037	18.635	18.651	0.016	2226 684	5234 647^a^
											
											
**No prophylaxis *vs* prophylaxis with raloxifene**	**No prophylaxis**	**Raloxifene**	**Incremental**	**No prophylaxis**	**Raloxifene**	**Incremental**	**No prophylaxis**	**Raloxifene**	**Incremental**	**(¥/LYG)**	**(¥/QALY)**
*Five-year predicted breast cancer risk ⩾1.66%*											
Starting at age 50	17 630 814	17 833 020	202 206	22.168	22.190	0.022	22.040	22.027	−0.013	9256 382	Cost more, gain less
Starting at age 60	20 160 906	20 427 386	266 480	18.806	18.822	0.016	18.688	18.670	−0.018	16 806 286	Cost more, gain less
											
*Five-year predicted breast cancer risk 3.01–5.00%*											
Starting at age 50	17 579 407	17 794 890	215 482	22.195	22.214	0.019	22.088	22.071	−0.017	11 599 422	Cost more, gain less
Starting at age 60	20 251 937	20 529 452	277 515	18.808	18.820	0.012	18.718	18.694	−0.024	23 845 594	Cost more, gain less
											
*Five-year predicted breast cancer risk ⩾5.01%*											
Starting at age 50	17 867 146	17 911 198	44 053	22.049	22.111	0.062	21.832	21.871	0.039	705 126	1123 880^a^
Starting at age 60	19 958 433	20 161 888	203 455	18.797	18.839	0.042	18.614	18.633	0.019	4848 677	10 664 954
											
*History of lobular carcinoma* *in situ*											
Starting at age 50	17 856 158	17 935 697	79 540	22.054	22.107	0.053	21.841	21.869	0.027	1496 425	2904 386^a^
Starting at age 60	19 968 466	20 186 549	218 083	18.798	18.833	0.036	18.618	18.628	0.010	6133 167	21462 765
											
*History of atypical hyperplasia*											
Starting at age 50	17 806 095	17 795 708	−10 387	22.079	22.156	0.077	21.884	21.942	0.058	Cost less, gain more	Cost less, gain more
Starting at age 60	20 015 243	20 198 328	183 085	18.800	18.852	0.052	18.635	18.668	0.033	3527 453	5570 154^a^

a Cost-effective when compared to a suggested criterion in Japan (Ohkusa, 2003) of ¥6000  000 for one QALY gain.

**Table 5:** Results of cost-effectiveness analysis (2)

	**Cost (¥)**			**Effectiveness (LYGs)**			**Effectiveness (QALYs)**			**Incremental cost-effectiveness ratio**	
**Prophylaxis with tamoxifen *vs* prophylaxis with raloxifene**	**Tamoxifen**	**Raloxifene**	**Incremental**	**Tamoxifen**	**Raloxifene**	**Incremental**	**Tamoxifen**	**Raloxifene**	**Incremental**	**(¥/LYG)**	**(¥/QALY)**
*Five-year predicted breast cancer risk ⩾1.66%*											
Starting at age 50	17 751 353	17 833 020	81 667	22.167	22.190	0.023	22.000	22.027	0.027	3501 723	3035 955^a^
Starting at age 60	20 324 294	20 427 386	103 093	18.807	18.822	0.015	18.654	18.670	0.016	7107 875	6364 920
											
*Five-year predicted breast cancer risk 3.01–5.00%*											
Starting at age 50	17 732 900	17 794 890	61 990	22.185	22.214	0.029	22.037	22.071	0.034	2163 079	1839 670^a^
Starting at age 60	20 444 141	20 529 452	85 312	18.797	18.820	0.023	18.666	18.694	0.028	3741 906	3063 477^a^
											
*Five-year predicted breast cancer risk ⩾5.01%*											
Starting at age 50	17 800 766	17 911 198	110 432	22.096	22.111	0.015	21.854	21.871	0.017	7150 490	6542 190
Starting at age 60	20 058 020	20 161 888	103 869	18.825	18.839	0.014	18.618	18.633	0.015	7476 332	6771 100
											
*History of lobular carcinoma* *in situ*											
Starting at age 50	17 850 772	17 935 697	84 925	22.085	22.107	0.022	21.843	21.869	0.025	3846 426	3359 650^a^
Starting at age 60	20 093 211	20 186 549	93 338	18.815	18.833	0.018	18.606	18.628	0.022	5064 724	4311 015^a^
											
*History of atypical hyperplasia*											
Starting at age 50	17 692 020	17 795 708	103 688	22.139	22.156	0.018	21.922	21.942	0.019	5922 294	5320 037^a^
Starting at age 60	20 096 731	20 198 328	101 598	18.837	18.852	0.015	18.651	18.668	0.017	6637 332	5872 017^a^

a Cost-effective when compared to a suggested criterion in Japan (Ohkusa, 2003) of ¥6000 000 for one QALY gain.
